# Distributed denial of service detection and mitigation in software-defined networking-enabled software-defined wide area networks

**DOI:** 10.1371/journal.pone.0346673

**Published:** 2026-05-12

**Authors:** Mohamed Musa, Tan Fong Ang, Yen-Lin Chen, Uzair Aslam Bhatti, Chin Soon Ku, Yu Luo, Jiahui Chen, Lip Yee Por

**Affiliations:** 1 Department of Computer System and Technology, Faculty of Computer Science and Information Technology, Universiti Malaya, Kuala Lumpur, Wilayar Persekutuan, Malaysia; 2 Department of Computer Science and Information Engineering, National Taipei University of Technology, Taipei, Taiwan; 3 School of information and Communication Engineering, Hainan University, Hainan, China; 4 Department of Computer Science, Universiti Tunku Abdul Rahman, Kampar, Perak, Malaysia; 5 School of Computer Science and Technology, Guangdong University of Technology, Guangzhou, China; Ajman University, UNITED ARAB EMIRATES

## Abstract

Software-defined wide area networks (SD-WAN), empowered by software-defined networking (SDN) technology, offer unparalleled flexibility and efficiency in wireless communication. However, their integration introduces new security challenges, particularly in mitigating distributed denial of service (DDoS) attacks. In this paper, we propose an advanced security framework tailored to SDN-enabled SD-WAN. A dataset collected during experiments was used for training and testing the model’s performance. Our framework leverages machine learning algorithms to detect and classify DDoS attacks targeting SD-WAN controllers, considering the environment’s unique characteristics. We develop adaptive machine learning model capable of accurately discerning high-rate and low-rate DDoS attacks, enhancing the network’s resilience against sophisticated threats. Results from controlled experiments show promise for real-world deployment, though further validation is needed. Our results highlight the framework’s ability to adapt to dynamic network conditions and provide robust security for SDN-enabled SD-WAN. Our adaptive model integrates RF and DT explicitly for SD-WAN contexts, achieving 99.97% accuracy for high-rate attacks and 99.96% for low-rate attacks. Our QT-PCA preprocessing pipeline reduces dimensionality while preserving performance, and PACKET_IN event-triggered mitigation enables dynamic response. This method significantly enhances security solutions’ accuracy, ensuring robust DDoS attack detection in SD-WAN environments. Additionally, by leveraging the SD-WAN architecture’s efficiency, our approach optimizes network performance, underscoring its efficacy and practicality in enhancing security and efficiency.

## Introduction

Wide Area Networks (WANs) find frequent applications in environmental monitoring systems, atmospheric monitoring systems, material sensing systems, security or surveillance systems, medical systems [1], and cyber-physical systems [2]. These networks leverage the collective computing capabilities of devices, which are determined by their physical sensing and processing capabilities [3]. Facilitated by the network control system, communication between devices is established, allowing for data processing and response. WANs open doors to applications in previously inaccessible environments [4].

The characteristic flexibility of WANs allows for the deployment of numerous devices across diverse regions, facilitated by communication among devices to access a WAN remotely; additionally, a network gateway may serve as an interface to connect to the Internet [5]. This phenomenon presents an opportunity for innovation in network computing.

Software-defined networking (SDN) represents a modern architecture that decouples logical control functions using the OpenFlow protocol for data-forwarding devices [6]. SDN architectures mark a significant advancement in computer networks, offering numerous benefits and complete visibility and control over network equipment, facilities, and applications [7]. This architectural innovation involves a three-tier structure comprising the infrastructure, control, and application layers, interconnected by two communication channels [8]. A controller is integrated into the network architecture to enable centralized network control [9]. However, the centralized control feature can lead to single-point failures [10], increasing vulnerability to distributed denial-of-service (DDoS) attacks [11].

SDN also facilitates a functional interface for sensor re-programmability, which is essential for function switching without requiring additional hardware [12]. Various types of DDoS attacks target the SDN controller, including User Datagram Protocol (UDP), Transmission Control Protocol (TCP/SYN) floods, Ping of Death attacks, Hypertext Transfer Protocol (HTTP) floods, and Internet Control Message Protocol (ICMP) floods [13]. While these attacks exploit different aspects of network protocols, our proposed method specifically addresses TCP/SYN and UDP flood attacks. We effectively mitigate the impact of these floods, ensuring that SDN controllers remain resilient against related common DDoS threats.

Software-defined wireless sensor networks (SD-WAN) represent a network computing paradigm applying SDN strategies to enhance WAN technological applications [14].

SD-WAN nodes operate at low rates and have limited processing power, making them vulnerable to saturation attacks that can lead to DDoS attacks; for instance, if the gateway between the SDN controller and the WAN features a restricted bandwidth radio module, this could render the controller a weak link despite having the resources to repel the attack [15].

The research literature reveals several pertinent observations regarding the suitability of existing datasets for evaluating SDN network performance. It has been noted that datasets such as NSL-KDD, KDD-cup99, ISCX, and CIC-IDS2017, although widely used, were primarily developed for conventional networks and lack comprehensive features relevant to SDN environments [16].

Ma et al. [17] conducted a study using two datasets: Rocketfuel [18] and CAIDA [19]. The study aimed to detect and mitigate DDoS attacks solely from data plans in SDN environments; this presents challenges due to limited visibility, resource constraints, scalability issues, and the need for centralized intelligence. Data planes are optimized for packet forwarding but lack the computational resources for sophisticated DDoS detection. Additionally, the sheer volume of traffic can overwhelm data plane processing. Effective detection and mitigation require centralized controllers to aggregate data, analyze traffic patterns, and orchestrate mitigation strategies based on evolving network conditions [20].

In the context of SD-WAN, leveraging deep learning (DL) models such as deep neural networks (DNNs) [21,22], and autoencoders [23,24] presents promising opportunities for handling high-dimensional and non-linear datasets effectively. However, it’s crucial to acknowledge the significant data requirements for training DL models, as they often demand large amounts of labeled data to achieve high accuracy [25]. This poses challenges in SD-WAN environments were collecting and labeling such datasets can be resource-intensive and may strain hardware resources, leading to increased energy consumption [26].

Ensemble models, such as k-nearest neighbors (KNN) and random forest (RF), offer flexibility and adaptability to various data types, capturing complex relationships even in the presence of noise or non-linear patterns [27]. Additionally, combining machine learning (ML) models with dimensionality reduction techniques like principal component analysis (PCA) can enhance accuracy while reducing computational overhead and energy consumption [28]. Preprocessing techniques such as quantile transformation can also improve the suitability of data for ML models by addressing non-normality, equalizing feature scales, and preserving relative distances [29]. Overall, in SD-WAN deployments, careful consideration of the benefits and challenges of DL approaches, alongside exploration of alternative techniques, is essential to ensure effective utilization of machine learning while meeting the constraints of energy efficiency and hardware resources.

Notably, none of the examined studies successfully applied machine learning techniques to discern high- and low-rate DDoS attacks, with concerns raised over the trade-offs between feature complexity, computational resources, and controller load. Hence, ensuring the accuracy of data inputs remains paramount for achieving reliable analytical outcomes. This work addresses the outlined challenges by presenting an integrated system capable of effectively detecting and mitigating various forms of DDoS attacks. The contributions of this study include:

- Develop detection mechanisms for DDoS attacks on SDN-enabled SD-WAN controllers.
- Design an adaptive dual-model to detect high-rate and low-rate DDoS attacks with minimal false positives.
- Design a modular, testable framework for SD-WAN DDoS detection and mitigation.
- Implementation of a closed-loop system integrating detection with proactive mitigation through SDN controllers.
- Implement mitigation strategies to reduce DDoS impact.
- Evaluate the method’s performance against various DDoS attacks.
- Assess detection accuracy and mitigation effectiveness in SDN-enabled SD-WAN environments.

The rest of this paper is divided into the following sections: related works in Section 2; Section 3 depicts the proposed methodology; and Section 4 illustrates the experimental design and results. Finally, Section 5 concludes this study.

## Related work

Mansoor et al. [30] introduced a deep learning-based approach for detecting DDoS attacks on a software-defined networking controller (DLADSC) by identifying distinctive features that differentiate DDoS network traffic from regular traffic. It comprises three stages: Data preprocessing involves preparing input data for further analysis by applying transformations and normalization techniques. Cross-feature selection identifies the most critical features. These selected features are then used to train an RNN model to capture temporal patterns and dependencies in network traffic, enabling the detection of DDoS attacks. The DLADSC approach achieved notable performance metrics: an average accuracy of 94.18%, precision of 92.14%, false positive rate of 8.11%, recall of 96.50% and an F1 score of 94.27%. However, it exhibits limitations in detecting low-rate attacks and scalability issues when dealing with large-scale network environments.

Chouhan et al. [31] proposed a method to detect DDoS attacks in SDN environments using feature extraction and classification on live traffic. The method emphasizes effective feature extraction to improve machine learning performance. Various classifiers were trained and tested, including SVM, Random Forest, K-Nearest Neighbor, XGBoost, and Naive Bayes. SVM performed best, achieving 99.39% accuracy, 99.41% precision, 99.39% recall, 0.71% FAR, 0.99 AUC, and 99.4% F1 score using a custom dataset with seven features. However, this method may not suit combined SDN and SD-WAN environments due to limited feature suitability and SVM’s potential lack of generalization to other network architectures.

One of the most often-used DDoS detection approaches is the threshold-based DDoS detection method proposed by Halman and Alenazi [32], which offers an efficient, low-complexity solution for mitigating DDoS attacks in healthcare networks. This method utilizes a threshold value of 57 bytes per packet to distinguish between attacks and normal samples, achieving an impressive accuracy of 99%. Evaluation metrics include accuracy, where TBDC attains 99% accuracy on collected data. The dataset used in this study consists of both normal and DDoS traffic generated within a healthcare virtualized network based on software-defined networking (SDN). However, a notable limitation of TBDC is its reliance on a fixed threshold value, potentially hindering its effectiveness in capturing the nuances of both high- and low-rate DDoS attacks in complex SDN-SD-WAN architectures. Thus, adapting TBDC to effectively combat both high- and low-rate DDoS attacks in combined SDN-SD-WAN environments may necessitate further exploration and refinements.

In the study by Fotse et al. [33], the CICDoS2017, CICDoS2019, and InSDN datasets were used to train a Federated Learning model under the Ryu Controller. The system achieved an accuracy of 98.55%, with PR, Recall, and F1 score of 98.21%, 98.31%, and 98.51%, respectively. This research introduced a DDoS attack detection system for large-scale SDN environments, leveraging federated learning for distributed detection. The model demonstrated promising efficiency in detecting DDoS attacks while reducing the load on individual controllers. However, challenges exist, particularly in safeguarding the system against data poisoning and improving model aggregation techniques. Future research should focus on enhancing aggregation strategies and countering adversarial ML threats to maintain robust detection capabilities.

Jafarian et al. [34] used NetFlow and OpenFlow features to train a Gradient Boosting Trees (GBT) model under the Floodlight controller. Their model, applied to a dataset of 157,500 records, achieved an accuracy of 98.8%, with PR, Recall, and F1 score values of 99.79%, 98.25%, and 98.99%, respectively. The study highlighted the challenges in SDN security, particularly regarding DDoS attack detection. They proposed an anomaly detection strategy that improves scalability and detection accuracy by using GBT, NetFlow, and IGR feature selection. The system outperformed existing methods, achieving an accuracy of 98.80% and reducing false alarm rates significantly. By integrating REST API technology in the Floodlight controller, the system also enabled the removal of malicious users, improving the overall network security. Future work should explore additional ways to mitigate control plane saturation and enhance scalability.

Mehmood et al. [35] combined CNN and MLP algorithms for DDoS detection on the CICDoS2019 and InSDN datasets, achieving outstanding results with an accuracy of 99.95% and PR, Recall, and F1 score of 99.90%, 99.97%, and 99.93%, respectively. Their novel approach, utilizing a Bayesian optimizer and Shapley Additive feature selection, significantly enhanced the effectiveness of DDoS detection. This study showed that the proposed CNN-MLP model outperforms existing detection systems, making it highly suitable for SDN environments. Their work contributes to DDoS detection research and presents potential avenues for future development, particularly in network security. Integrating Bayesian optimization and feature selection techniques could be a promising direction for improving the accuracy and efficiency of detection models.

Wang et al. [36] proposed a method called Multi-dimensional Traffic Characteristics (MDDCC) to enhance intrusion detection in SDN environments. This approach utilizes switch statistics to first narrow down potential threats before applying DL techniques for a detailed analysis, thereby increasing overall detection accuracy. The method leverages multiple datasets, including InSDN, CIC-IDS2017, and CIC-DDoS2019, demonstrating impressive performance metrics with an accuracy of 99.24%, precision of 98.00%, recall of 99.68%, and an F1 score of 99.33%. Additionally, on CIC-IDS2017, the method achieved an accuracy of 99.59% and an F1 score of 99.65%, showcasing its robustness across various datasets.

However, the substantial computational resources required for tasks such as wavelet decomposition, Convolutional Neural Network (CNN) processing, and classification can overwhelm the controller, especially when a high-rate attack is flooded, creating a heavy processing burden. Misjudgments in the initial threat assessment phase may further strain the controller by imposing unnecessary loads in subsequent stages, potentially leading to missed detections. Thus, further exploration is needed to optimize the MDDCC framework for improved efficiency.

Our proposed method applied a feature extraction technique to a dataset containing 232,714 records, achieving perfect precision, recall, and F1 score of 99.99%. The model, trained using algorithms such as Decision Trees (DT), KNN, Naive Bayes (NB), RF, and SVM under the Ryu Controller, achieved an accuracy of 99.97%. This approach considered both low- and high-rate attacks, demonstrating exceptional performance in detecting DDoS threats. Multiple algorithms and feature extraction methods contributed to the model’s high accuracy and robustness. Integrating diverse machine learning algorithms enhances the system’s ability to identify various types of attacks in SDN environments. The S1 Table in the supplementary documents shows the comparative analysis with other methods.

## Incorporating hybrid evolutionary methods and sensitivity analysis

To broaden the methodological perspective of DDoS detection research, recent studies have explored hybrid evolutionary algorithms and sensitivity analysis for feature optimization and model interpretability. Sharghi et al. [37] propose a hybrid Random Forest–Genetic Algorithm (RF-GA) framework for forecasting groundwater levels under climate change, emphasizing input delay optimization and variable sensitivity analysis. Their results demonstrate how evolutionary algorithms can enhance predictive accuracy and robustness in dynamic systems. Markovic et al. [38] apply genetic algorithm–based feature selection for intrusion detection on resource-constrained edge devices, highlighting the importance of balancing computational efficiency and detection performance in SDN-like environments. Panigrahi et al. [39] present an explainable AI framework that uses sensitivity analysis to quantify the robustness and explanatory power of machine learning models. This approach informs feature importance assessment and preprocessing strategies, which can directly benefit DDoS detection models. Building on these insights, our Future Work section now discusses:

- the potential of hybridizing evolutionary algorithms for feature optimization.
- the application of sensitivity analysis to improve model interpretability and adaptive capabilities in dynamic SDN-SD-WAN environments.

## Materials & methods

**Fig 1:**
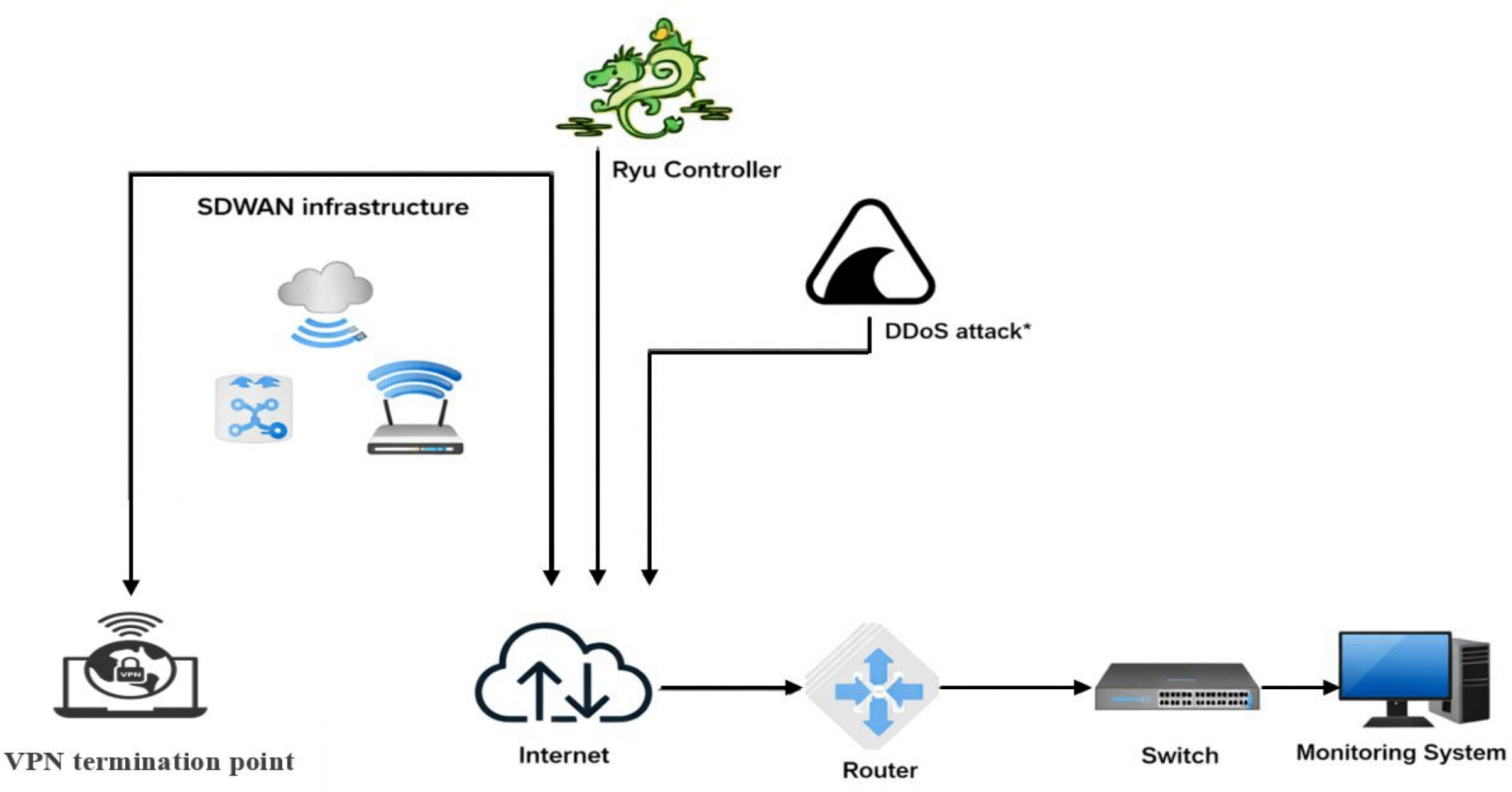
The proposed SD-WAN architecture.

Fig 1 illustrates a comprehensive SD-WAN infrastructure designed to efficiently manage network traffic while mitigating potential threats such as DDoS attacks.

At its core lies the SDN controller, which facilitates centralized network management and control. Edge devices and gateways are interconnected with the WAN, providing access to the internet and other remote resources. Real-time network traffic monitoring is enabled through sFlow-RT [40], while Grafana offers visualization for performance analysis. Together, these components form a robust SD-WAN framework capable of optimizing connectivity and ensuring network security. This integrated approach empowers organizations to enhance productivity and reliability across their distributed networks.

**Fig 2:**
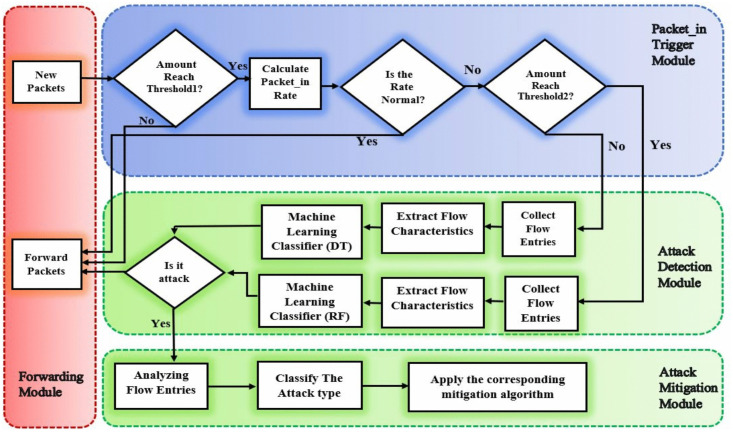
Multi-module DDoS defense strategy.

While our method addresses limitations identified in prior studies, it also extends beyond the scope of resolving a singular methodological challenge. By introducing a comprehensive DDoS protection and monitoring mechanism, our work contributes to the advancement of cybersecurity within SDN-enabled SD-WAN environments. As depicted in Fig 2, we have developed a multi-faceted approach comprising packet_in triggering, detection, mitigation, packet forwarding, and monitoring modules. This holistic framework enables effective detection and mitigation of high- and low-rate DDoS attacks, addressing critical security concerns in modern network infrastructures. Next, we will take a closer look at these modules.

### Packet in the trigger module

Detecting and defending against DDoS attacks presents significant challenges due to the periodic detection trigger techniques commonly employed by systems. When an attack occurs, it places immense loads on controllers and switches.

This research introduces an anomaly detection approach named PACKET IN as a platform detection trigger mechanism to address the aforementioned challenges. When the SD-WAN controller receives a PACKET IN message, it extracts the complete original data packet to retrieve the packet’s header. Subsequently, based on the parsed result, a new flow record is constructed, and the relevant header matching field is filled in the record. The number of packets, PACKET_COUNT, is then set to 1, and the byte count, BYTE_COUNT, is set as the packet size.

Whether a DDoS attack is initiated through IP spoofing can be determined by monitoring the generation and transmission of a large number of PACKET_IN messages to the controller. The method begins by initializing several variables. Firstly, the Windows value is set to a predefined number of PACKET_IN, and the “speed” threshold is set to a sufficiently large value. The PACKET_IN count variable is utilized to track the number of PACKET_IN messages received by the controller. Modular arithmetic is performed using Windows values and PACKET_IN counts. If the result of the computation is zero, the current time (Tnow) is recorded, and the time interval ΔT between the current time (Tnow) and the initial time (Tpre) is calculated. Otherwise, the controller is alerted that the PACKET_IN should be processed. The window value is then divided by the time interval ΔT to obtain the PACKET_IN speed rate.

A low-rate attack is flagged if the PACKET_IN speed exceeds rate Threshold1; conversely, if the PACKET_IN speed surpasses rate Threshold2, a high-rate attack is noted. Otherwise, the controller is informed that the PACKET_IN should be processed. The stages of the algorithm are detailed in Algorithm 1.

**Algorithm 1. PACKET_IN Message Anomaly Detection**

**Input**: PACKET_IN messages

**Out**: Is the PACKET_IN message abnormal?

Initialize Windows_value, Speed_threshold1, Speed_threshold2

If a PACKET_IN message arrives, then

 PACKET_IN_count = PACKET_IN_count + 1;

 If PACKET_IN_count mod Windows_value == 0 then

  Record the time t_now_;

  ΔT = t_now_ − t_pre_;

  PACKET_IN_speed = Windows_value/ΔT;

Else

  Notify the controller to process PACKET_IN messages;

End if

If PACKET_IN_speed > Speed_Threshold1 then

  Return low-rate abnormal traffic alarm;

Else If PACKET_IN_speed > Speed_Threshold2 then

  Return high-rate abnormal traffic alarm;

 End If

Else

 Notify the controller to process PACKET_IN messages;

End If

### Attack detection module

This module integrates the best-performing algorithms for low-rate and high-rate attack detection within the SDN controller. This dual-algorithm approach ensures comprehensive protection, leveraging the strengths of each algorithm to address the distinct characteristics of low-rate and high-rate DDoS attacks.

### Adaptive dual-model detection framework

The proposed system adopts an adaptive dual-model detection framework rather than a conventional ensemble approach. Instead of executing multiple classifiers simultaneously, only one model is activated at a time based on real-time traffic characteristics observed at the SDN controller. A lightweight traffic characterization module continuously monitors two indicators: (i) the PACKET_IN event rate (packets per second) and (ii) the SYN-to-total packet ratio.

Model selection is context-aware. High-rate or SYN-dominant traffic triggers the Decision Tree (DT) due to its low inference latency, while moderately anomalous traffic activates the Random Forest (RF) to capture more subtle attack patterns. Normal traffic bypasses the detection models to avoid unnecessary computation. The models are trained on attack-specific subsets and never run concurrently. A confidence-based fallback mechanism invokes the alternate model only when prediction certainty is low.

This design minimizes computational overhead while maintaining accurate detection across both high-rate volumetric attacks and stealthy low-rate attacks, making it suitable for real-time SDN controller deployment.

### Attack differentiation mechanism

The framework distinguishes between high-rate and low-rate DDoS attacks through two complementary mechanisms.

1. Feature-Level Basis: Analysis of the CIC-DDoS2019 dataset reveals distinct statistical signatures. High-rate attacks exhibit extremely high packet rates, short flow durations, and >90% SYN flags. Low-rate attacks show moderate packet rates, longer flows, and balanced flag distributions (40–60% SYN).
2. Architecture-Level Mechanism: A lightweight monitoring module evaluates real-time traffic metrics (packet rate, flag ratios) and dynamically activates the appropriate classifier:

- DT is activated for sudden volumetric spikes. High-rate attacks produce sharply separable thresholds, making DT’s rule-based splitting both accurate and computationally efficient for rapid mitigation.
- RF is activated for moderate-rate anomalies. Low-rate attacks manifest as subtle multivariate patterns where RF captures nonlinear interactions, reducing false positives while maintaining sensitivity.

This context-aware specialization enables both rapid detection of high-rate floods and sensitivity to stealthy low-rate attacks.

### Collect flow entries

Upon detecting an attack trigger, the collection of flow table entries is initiated, providing vital data for DDoS detection within SD-WAN. Key features extracted for model training are listed in Table 1, including packet count and byte count, which monitor traffic volume to identify sudden spikes indicative of potential attacks.

**Table 1:** The extracted features and their descriptions.

Feature name	Features description
time_stamp	The current timestamp for an event
data_path_id	Datapath ID for the arrived traffic
flow_id	Unique ID for arriving traffic
ip_source	Packet Source IP address
tp_source	Packet Source Port address
ip_dstn	The destination IP address
tp_dstn	Destination Port address
ip_proto	Protocol type of the IP address
icmp_code	first byte from the ICMP message.
icmp_type	Second byte from the ICMP message.
flow_dur_sec	Duration of the flow per second
flow_dur_nsec	Duration of the flow per nanosecond
idl_time_out	Validity of entry in seconds.
hd_time_out	Absolute timeout after blocking a flow
flags	Indicate the physical port behavior.
pkt_count	The rate at which packets receive
byte_count	The rate at which bytes are received
pkt_count_sec	Total packets arrived per second
pkt_count_nsec	Total packets arrived per nanosecond
byte_count_sec	Number of bytes arrived per second
byte_count_nsec	Number of bytes arrived per nanosecond

Flow duration is used to discern anomalies in flow behavior, which is crucial for distinguishing between legitimate traffic and malicious activity. Additionally, IP Source and Destination, along with Port Address, offer insight into the origin and destination of network traffic, facilitating the pinpointing of suspicious activity within the SD-WAN infrastructure. These tailored features are designed to address the dynamic nature of SD-WAN environments.

To mirror real-world DDoS traffic patterns, we intentionally introduced class imbalance in the dataset by extending the duration of attack flows during data collection. Using hping3 [41], we generated attack traffic for significantly longer periods (72% of total collection time) compared to legitimate traffic (28%), resulting in a representative distribution of 232,714 instances: 98,970 legitimate (42.5%), 83,320 low-rate DDoS (35.8%), and 50,424 high-rate DDoS (21.7%). The final evaluation used a held-out test set (25% of 232,714 records). This temporal imbalance was achieved through controlled alternation between (1) short bursts of legitimate traffic generation and (2) sustained attack periods with varied parameters (packet rates: 50–10,000 pps, sizes: 64–1500 bytes, protocols: TCP/UDP/ICMP). For example, to generate high-rate DDoS traffic, we used the following hping3 command: hping3 --flood -p 80 --tcp --rand-source target_ip. This command sends a flood of TCP packets to port 80 on the target IP, simulating a high-rate DDoS attack. The ground truth labeling combined IP reputation checks, flow-rate thresholds, and header anomaly detection.

The resulting dataset’s intentional skew (57.5% attack traffic) provides a more authentic testbed for evaluating detection systems against operational conditions where attack volumes typically overwhelm normal traffic. Given the inherent class imbalance, we applied class weighting during model training to mitigate bias toward the majority class. However, instead of using oversampling or undersampling techniques, class weights were automatically calculated based on inverse class frequencies. This approach allowed the models to emphasize minority class samples without generating synthetic data, preserving the original traffic distribution and reducing the risk of overfitting. Class weighting was consistently applied across all classifiers to ensure balanced learning in our imbalanced dataset.

To assess the realism of our simulated attack patterns, A quantitative comparison of our simulated attacks and widely used real-world benchmark datasets including CICDDoS2019 [42] and inSDN [43], is provided in S3 Table. These datasets consist of labelled traffic captured in controlled testbed environments that emulate enterprise network conditions rather than live operational SD-WAN deployments. The comparison evaluates key statistical properties, including packet rates, flow durations, protocol distributions, SYN-flag ratios, and temporal burstiness, demonstrating that the simulated attacks closely reproduce the primary characteristics of volumetric and protocol-based DDoS events.

Our dataset includes representative attack types such as SYN floods, UDP floods, and HTTP application-layer floods, covering both high-rate and moderate-rate behaviors commonly reported in practice. However, real-world DDoS campaigns exhibit substantially greater heterogeneity and complexity. Operational SD-WAN environments often involve multi-vendor infrastructures, diverse background workloads, and a high prevalence of encrypted VPN/IPsec traffic, which limits visibility into payload-level features. Additionally, adversaries may employ multi-vector strategies, adaptive evasion techniques, low-and-slow attack patterns, and time-varying behavior that are difficult to reproduce in controlled simulations.

These factors can introduce domain shift when models trained on benchmark or emulated data are deployed in production networks, potentially altering feature distributions and reducing detection performance. Therefore, while the strong results reported in this study demonstrate proof-of-concept effectiveness under standardized conditions, real-world performance may degrade without periodic retraining, domain adaptation, or validation using operational telemetry. This limitation is explicitly acknowledged in the manuscript, and future work will focus on evaluating the framework on real SD-WAN traffic, incorporating encrypted-flow features, and extending the attack model to include more sophisticated multi-vector and stealth scenarios.

### Data preparation

The original dataset contained 34 network traffic features. Feature selection was performed using a combination of domain knowledge and tree-based importance rankings, resulting in a subset of 21 informative features. The dataset was then split into 80% training and 20% held-out testing sets. PCA and quantile transformation were applied for dimensionality reduction and scaling. A 20-fold cross-validation was performed on the training set for hyperparameter tuning and validation, with the test set kept completely separate. The final model was trained on the full training set and evaluated on the test set. This process was repeated over 20 independent runs with different random seeds, and average performance metrics with standard deviations are reported.

### Dimensionality reduction using PCA

**Fig 3:**
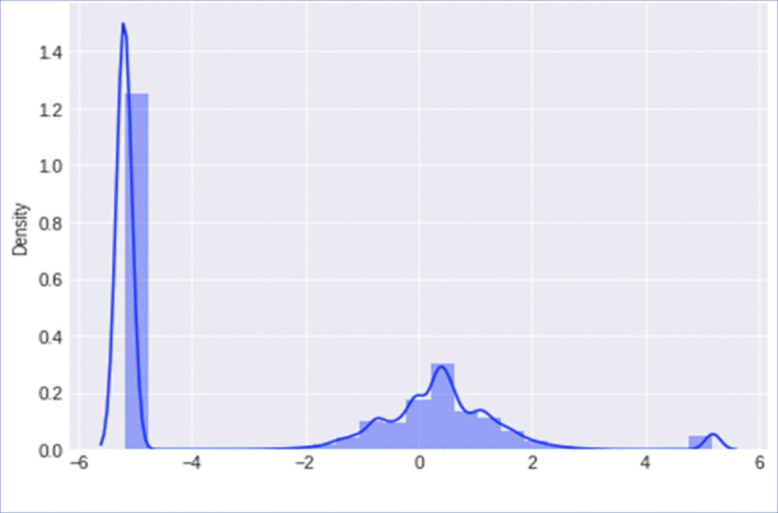
Multi-module DDoS defense strategy.

Dimensionality reduction involves projecting data into a lower-dimensional space by eliminating some input variables through dimensionality reduction techniques. The collected data in this study contains redundant, irrelevant, and noisy data, which can affect the data analysis task [44]. PCA is utilized to enhance the distribution of data and elevate its quality. Fig 3 depicts the original data distribution.

The mathematical model for PCA involves the following steps

1. Take the entire dataset with d+1 dimensions and remove the labels, resulting in a d-dimensional dataset. Calculate the mean of each dimension over the entire dataset.

$$\text{Mean of matrix A }=\overset{―}{A}$$ (1)

2. Compute the variance-covariance matrix using:

$$cov(X,Y)=\text{1}/n-\text{1}\sum_{i=\text{1}}^{n}\left(\left(x_{i}-\overset{―}{x}\right)\left(y_{i}-\overset{―}{y}\right)\right)$$ (2)

3. Calculate Eigenvectors and their associated Eigenvalues. Let A be a square matrix, ν a vector, and λ a scalar satisfying Aν=λν. The eigenvalues of A are the roots of the characteristic equation:

det (A−λI) (3)

where I is an identity matrix.

4. Sort the eigenvectors by decreasing eigenvalues and choose the k eigenvectors with the most significant eigenvalues to construct a d*k dimensional matrix W.
5. Form the principal components using the formula:

$$\text{NewData }={\text{FeatureVector}}^{T}\times {\text{ScaledData}}^{T}$$ (4)

Here, NewData is the matrix created by the principal components, FeatureVector is the matrix generated by the eigenvectors, and ScaledData represents the scaled version of the original dataset. The letter ‘T’ denotes the transpose of a matrix, generated by swapping rows and columns.

To determine the optimal number of principal components, we evaluated the cumulative variance explained by different numbers of components. Using 3 components retained 85.7% of the total variance, while 5 components captured 92.4%. Increasing the number of components further (e.g., 7 or 10) only marginally increased variance retention (96.1% and 98.3%, respectively) but added computational overhead. Therefore, 5 principal components were selected as a trade-off between preserving most of the data’s information and maintaining computational efficiency for real-time SD-WAN traffic analysis. The transformation is performed using the fit_transform(X) method, where X represents the features of the dataset (all columns except the target variable). The resulting data, dt, now has 5 columns, each representing a principal component, but retains the same number of rows as the original dataset.

### Change data distribution

A quantile transform (QT) converts the probability distribution of one variable into another. The quantile function ranks or smoothes the relationship between observations and can be transferred to different distributions, such as uniform or normal [45]. QT can be performed using the following steps:

- Sort the data to estimate the cumulative distribution function.
- The QT of data in the input range corresponds to the cumulative distribution function (CDF) value.
- The boundaries of the CDF provide the QT for data outside of the input range.

**Fig 4:**
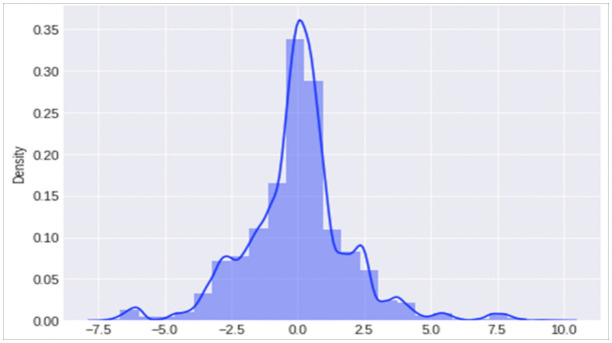
Data distribution after applying QT-PCA.

Fig 4 illustrates the data distribution after applying QT to the targeted data. QT is applied to the transformed data (dt) to normalize it. The n_quantiles = 15 parameter divides the data into 15 equal-sized bins based on the values, and the random_state = 0 ensures the process is reproducible. By setting output_distribution = ‘normal’, the transformation forces the features to follow a normal distribution (Gaussian distribution), where each feature has a mean of 0 and a standard deviation of 1. This transformation is beneficial when preparing data for algorithms that assume normally distributed inputs, as it can improve model performance. The transformed data, scaled_dt, now has the same number of rows as dt but with features that are normally distributed.

To validate the contribution of our preprocessing pipeline, we conducted an ablation study comparing four configurations: (i) raw features, (ii) PCA only, (iii) QT only, and (iv) QT followed by PCA (proposed). The results, presented in Supplementary S7 Table, demonstrate that applying PCA directly to raw features yields inferior performance due to distributional distortions, while QT alone improves robustness but retains higher dimensionality and computational cost. The combined QT → PCA pipeline achieves the optimal balance between detection accuracy, stability, and efficiency, making it suitable for real-time SD-WAN deployment

### Machine learning algorithms

The output of QT-PCA is used as input for the developed ML models. Table 2 describes a diverse array of machine-learning algorithms that were utilized to fulfill the research objectives. These included decision trees, K-nearest neighbors, support vector machines, random forests, and naive Bayes. The selection of each algorithm was meticulous, taking into account factors such as the data’s characteristics, the desired outcomes, and the specific analytical requirements. This deliberate choice facilitated a thorough exploration and analysis, bolstering the robustness and effectiveness of the research methodology. We selected Random Forest due to its robustness to overfitting and interpretability and XGBoost for its strong performance on tabular data and efficiency in handling high-dimensional features. These models were particularly well-suited to the SDN-enabled SD-WAN framework due to their ability to balance detection accuracy and computational cost.

**Table 2:** Machine learning algorithms and parameters utilized in deployment.

ML Algorithm	Parameters
Decision Tree (DT)	Criterion = entropy,min_samples_split = 2 min_samples_leaf = 1, Random state = 0
K-Nearest Neighbor (KNN)	n_neighbors=5, metric=‘minkowski’,p=2
Support Vector Machine (SVM)	kernel=‘rbf’, random_state=0
Random Forest (RF)	n_estimators=10, criterion=“entropy”, random_state=0
Naive Bayes (NB)	priors=None, var_smoothing=1e-09
ML Algorithm	Parameters
Decision Tree (DT)	Criterion = entropy,min_samples_split = 2 min_samples_leaf = 1, Random state = 0

Moreover, ensemble models like KNN were incorporated for their adaptability to various data types and their capability to discern complex relationships, even amidst noisy or non-linear patterns. Furthermore, the integration of ML models with dimensionality reduction techniques such as principal component analysis (PCA) showed promise in enhancing accuracy while mitigating computational burden and energy consumption. For production deployment, we performed Bayesian optimization with 20-fold nested cross-validation to refine hyperparameters. The final deployed models used RF (n_estimators = 150, max_depth = 25) and DT (min_samples_split = 5), while other parameters remained as default. This optimization improved the F1 score by 2.3% without increasing computational overhead.

Additionally, preprocessing techniques like quantile transformation played a pivotal role in refining data suitability for ML models by addressing issues such as non-normality and disparate feature scales. These alternative approaches present practical solutions for optimizing the utilization of machine learning in SD-WAN deployments, particularly in navigating the challenges posed by energy efficiency and hardware constraints.

### Attack mitigation module

Upon detection of a DDoS attack, the SDN controller initiates a dynamic mitigation process to limit the impact on network resources and maintain service availability. Unlike static blocking approaches, the proposed framework applies adaptive mitigation actions using OpenFlow control mechanisms based on real-time traffic characteristics. When anomalous flows are identified, the controller extracts the suspected source information from the Flow Stat Database (FSD), which stores flow-level statistics collected via SFlow. The mitigation logic leverages SFlowRT to process real-time measurement datagrams and generate summarized telemetry exposed through a northbound REST API. This enables rapid response to abnormal traffic patterns triggered by elevated PACKET_IN event rates. Algorithm 2 outlines the general process for mitigating UDP- and Transmission Control Protocol (TCP)-based DDoS attacks.

**Algorithm 2** **Attack Mitigation**

**Input**: Real-time measurement datagrams from SFlow.

**Output**: Flow entries installed on OpenFlow Switch.

Initialize Flow Stat Database (FSD), SFlowRT, and OpenFlow Switch.

While the network is running:

 Receive real-time data from SFlow.

 Process data with SFlowRT.

 Identify anomalous flows indicating DDoS attacks.

 If DDoS attack is detected:

  Extract the attacker’s IP address and flow characteristics from FSD.

  Determine attack intensity (high-rate or low-rate).

  Install mitigation rules via OpenFlow:

   Apply priority-based drop rules for confirmed malicious sources.

   Apply meter-based rate limiting for suspicious traffic.

   Configure hard and soft timeouts for rule lifecycle management.

  EndIf

EndWhile

### Mitigation actions via openflow control

The controller installs appropriate flow rules on the OpenFlow switch using FLOW_MOD messages. Depending on attack intensity and confidence level, the following actions may be applied:

- Priority-based blocking: High-priority drop rules are installed to discard packets originating from confirmed malicious sources.
- Meter table rate limiting: Suspicious traffic can be throttled instead of fully blocked, preserving legitimate flows during uncertain conditions.
- Hard timeout configuration: Temporary blocking rules automatically expire after a predefined interval, allowing recovery once the attack subsides.
- Soft timeout configuration: Rules are removed when matching traffic ceases, preventing stale entries from occupying switch resources.

These mitigation actions are dynamically adapted according to the detected attack characteristics:

- High-rate (volumetric) attacks: Immediate blocking or aggressive rate limiting is applied to prevent link saturation and controller overload.
- Low-rate or stealth attacks: Selective rate limiting or lower-priority filtering is used to reduce false positives while maintaining normal service availability.

This differentiated handling enables effective protection against both large-scale flooding attacks and subtle low-and-slow threats.

### Closed-loop detection–mitigation operation

The proposed approach implements a closed-loop workflow in which monitoring, decision-making, and enforcement occur continuously during network operation. Real-time telemetry from SFlow and PACKET_IN events informs mitigation decisions, while OpenFlow rules enforce countermeasures directly in the data plane. This design enables rapid response to evolving attacks while minimizing disruption to legitimate traffic. The integration of adaptive mitigation based on attack type (as described in mitigation actions via OpenFlow control section) ensures that the closed-loop operation remains both efficient and context-aware.

### Packet forwarding module

This module is solely responsible for bidirectionally forwarding network traffic between switches and the controller.

### Packet forwarding module

**Fig 5:**
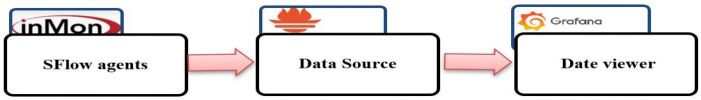
Overview of the monitoring process.

Our monitoring process, as illustrated in Fig 5, consists of a node exporter integrated with SFlow-RT, which is Real-time analytics coupled with scalable monitoring through embedded industry-standard sFlow instrumentation, fuels actionable intelligence for orchestration and SDN applications; the sFlow agents send traffic data and energy consumption statistics to the controller to monitor the network’s overall energy usage, while Prometheus [46] served as a data producer to convey the required data for visualization from the virtual machine, which generated both normal and abnormal traffic and pushed it to the data source, and Grafana retrieves data from the data source to present the desired information on the Grafana dashboard, visualizing it in two main formats: metrics and logs, with metrics including CPU load, current memory usage, and CPU temperature, and logs employed for troubleshooting purposes.

## Results and discussion

The experiments were conducted using an HP laptop equipped with an Intel Core i7-1065G7 processor running at 1.5 GHz and 16 GB of RAM, operating on Ubuntu Linux 20.04. Google Colab [47] was utilized for both training and testing purposes, offering 12 GB of RAM and a K80 processor. Mininet [48] served as the network emulator, while an OpenFlow Switch was employed as the switching device to establish a functional testbed. The Ryu V.4.43 [49] controller was used as the SDN controller.

## Results

The proposed approach is evaluated using standard metrics: detection accuracy (A), PR, recall, and F1 score. The confusion matrix demonstrates the values of the previously defined evaluation metrics:


$$A=\frac{\text{Accurately Classified Records}}{\text{Total Samples}}\times \text{1}\text{0}\text{0}$$ (5)

$$PR=\frac{\text{True Positive}}{\text{True Positive}+\text{False Positive}}\times \text{1}\text{0}\text{0}$$ (6)

$$R_{c}=\frac{\text{True Positive}}{\text{True Positive}+\text{False Negative}}\times \text{1}\text{0}\text{0}$$ (7)

$$F\text{1}=\text{2}\times \frac{\text{Rc}\times \text{Pr}}{\text{Rc}+\text{Pr}}\times \text{1}\text{0}\text{0}$$ (8)

**Fig 6:**
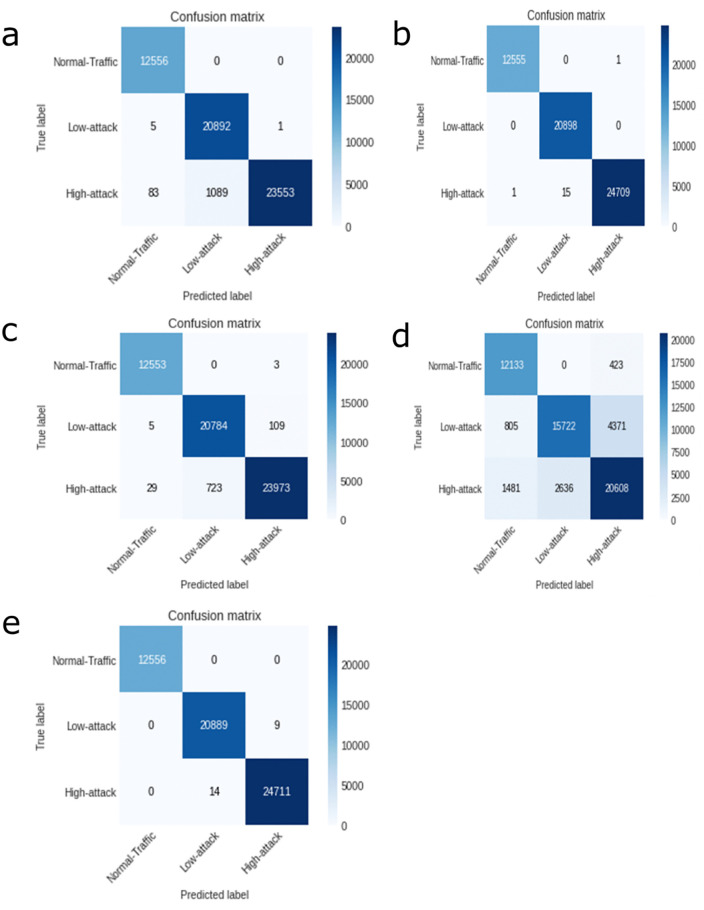
Confusion Matrices for Proposed ML Models. (a) SVM, (b) RF, (c) KNN, (d) NP, and (e) DT.

**Fig 7:**
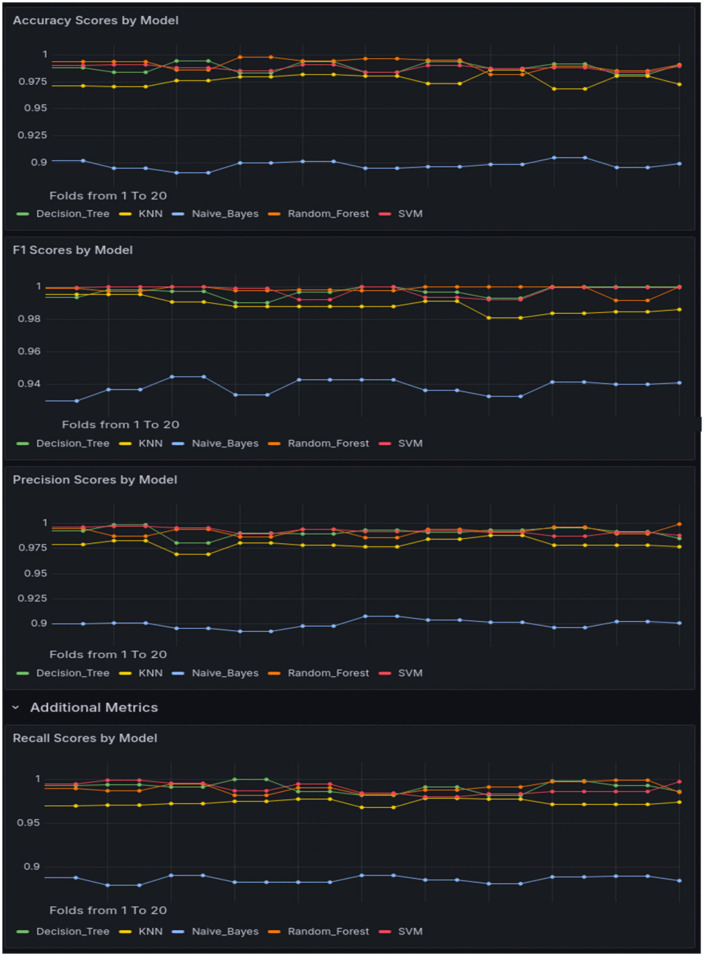
Evaluation metrics of proposed ML models.

Fig 6 displays the confusion matrices for each of the proposed ML algorithms (DT, KNN, NP, RF, and SVM). To improve result interpretability, we provide numerical confusion matrices in S6 Table, which details exact class-wise counts for True Positives (TP), False Positives (FP), and False Negatives (FN) across all evaluated models (SVM, RF, KNN, NB, and DT). Fig 7 illustrates the detection accuracy, weighted precision, recall, and F1 score of the suggested ML models, determined by taking the central diagonal values in the confusion matrix over the total number of samples.

### Analysis and discussion

Standard deviations, computed over 20 independent runs, are reported for accuracy, F1 score, recall, precision, and ROC-AUC to quantify performance. To ensure robustness, two distinct network topologies were evaluated across different configurations and attack scenarios, with the victim and attacker referred to interchangeably as Node 1 and Node 2. The results were further validated in terms of energy savings. Overfitting was mitigated through a 20-fold cross-validation procedure applied exclusively to the training set, feature selection combined with PCA for dimensionality reduction, and repeated experiments with different random seeds, with averaged results and corresponding standard deviations reported.

### Statistical comparison of model performance

We conducted paired statistical tests to compare the performance of various classifiers across multiple metrics, including AUC, accuracy, recall, precision, and F1 score, Statistical significance of model differences is quantified in S5 Table. The Random Forest classifier consistently outperformed Decision Tree, SVM, and Naive Bayes models with statistically significant improvements in all metrics (p < 0.05). For example, the Random Forest showed a mean AUC increase of 0.0004 (±0.0002) over the Decision Tree (p = 0.038, paired t-test) and a more pronounced gain of 0.0881 (±0.0051) over Naive Bayes (p < 0.001, Wilcoxon test). Similar significant differences were observed between the Decision Tree and SVM, as well as between the Decision Tree and KNN classifiers. These results demonstrate the superior predictive performance of Random Forest in our evaluation setting.

**Fig 8:**
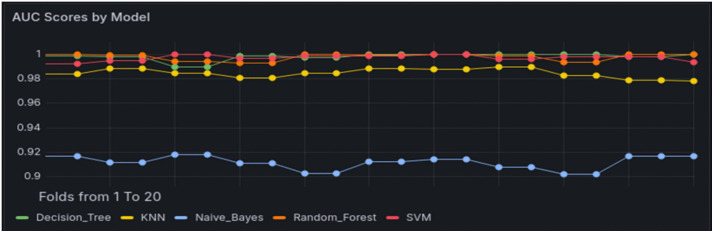
AUC-ROR for all ML models.

Given the inherent class imbalance in our dataset (57.5% attack vs. 42.5% benign), we applied class weighting during model training to mitigate potential bias toward the majority class. This approach assigns higher penalty weights to the minority class, helping the model better learn distinguishing features. Additionally, as presented in Fig 8, we incorporated the area under the receiver operating characteristic curve (AUC-ROC) as a key evaluation metric to better capture the models’ discriminative performance in this imbalanced context. The inclusion of AUC-ROC alongside accuracy, precision, recall, and F1 score provides a more robust assessment of model effectiveness.

**Fig 9:**
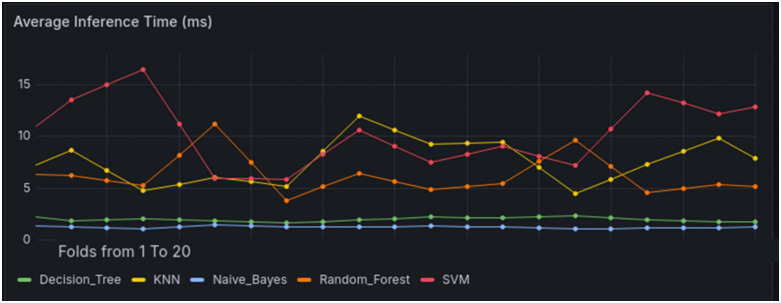
Inference Latency Comparison of ML Models.

As shown in Fig 9, the inference time metrics further validate our model deployment strategy. The Decision Tree consistently demonstrates the lowest latency (<5 ms), making it ideal for low-rate attack detection where rapid response is critical. The Random Forest, while slightly higher in latency (~10 ms), remains within real-time constraints and provides the accuracy needed for high-rate attack scenarios. In contrast, SVM exhibits prohibitively high inference times (~15 ms), and KNN shows variable latency (5–10 ms), rendering them unsuitable for time-sensitive SDN operations. Though Naive Bayes offers competitive speed (~5 ms), its exclusion—despite this advantage—aligns with our priority of balancing both timeliness and detection reliability. These measurements systematically justify our selection of the Decision Tree and Random Forest models based on their optimal trade-off between speed and performance.

**Fig 10:**
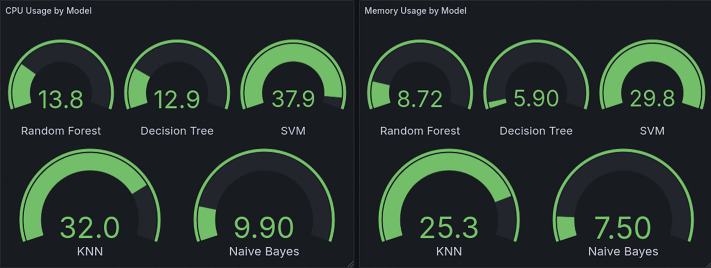
Percentage CPU and Memory Utilization.

As illustrated in Fig 10, our model deployment choices are grounded in empirical CPU and memory usage data. The Decision Tree (CPU: 12.9%, memory: 5.90%) and Random Forest (CPU: 13.8%, memory: 8.72%) exhibit the lowest resource consumption among the evaluated models, aligning with their roles in low-rate and high-rate attack detection, respectively. In contrast, the SVM (CPU: 37.9%, memory: 29.8%) and KNN (CPU: 32.0%, memory: 25.3%) impose significantly higher computational overhead, making them impractical for real-time SDN deployment. While Naive Bayes (CPU: 9.90%, memory: 7.50%) is the most resource-efficient, its exclusion—despite these advantages—was necessitated by its inferior detection accuracy as detailed in the previous section. This data-driven approach ensures an optimal balance between operational feasibility and detection efficiency.

**Fig 11:**
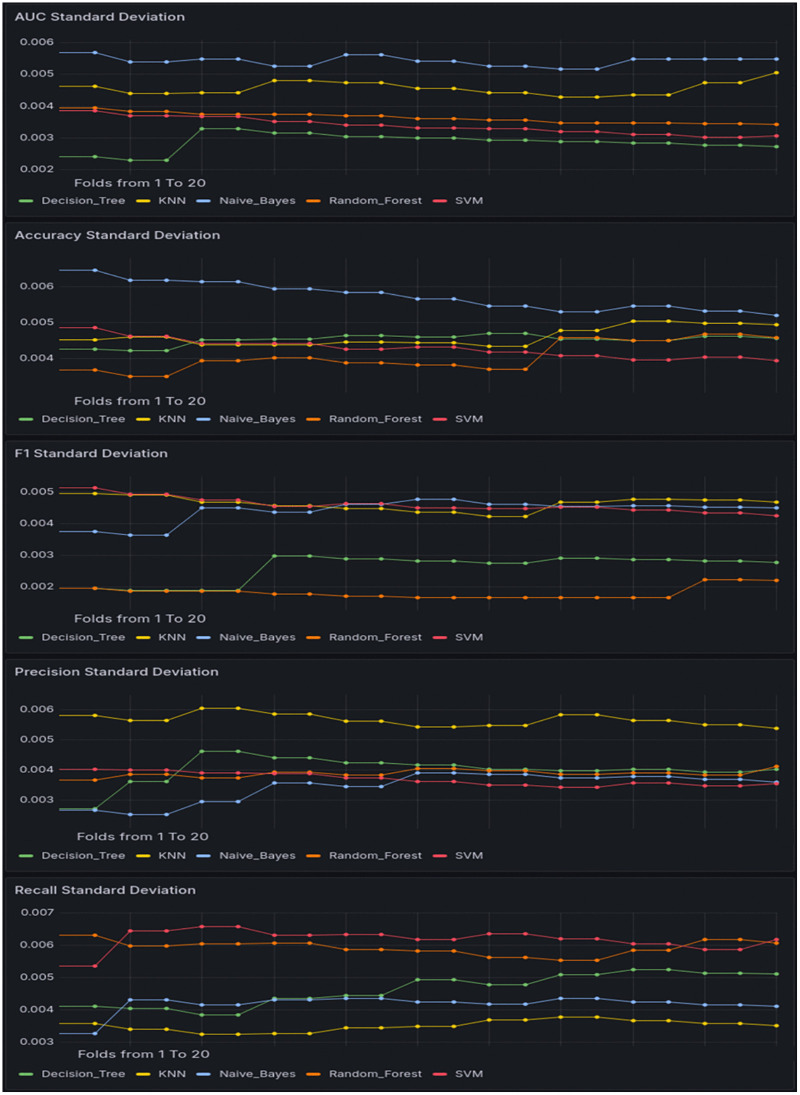
Standard Deviation Trends of Performance Metrics Across Machine Learning Classifiers.

Fig 11 illustrates the temporal variation in the standard deviation (SD) of five key performance metrics—AUC, Accuracy, F1 score, Precision, and Recall—across five machine learning classifiers: Decision Tree (DT), KNN, Naïve Bayes (NB), Random Forest (RF), and SVM. For AUC SD, NB consistently exhibited the highest variability (~0.005–0.006), followed by KNN (~0.004–0.005), while DT maintained the lowest (~0.002–0.0028) with a slight decreasing trend. In Accuracy SD, NB again showed the largest fluctuations (~0.0055–0.0064), whereas RF recorded the smallest values (~0.0035–0.0042) with minimal variation. F1 score SD trends were more uniform across models, with most classifiers ranging from 0.004 to 0.005, except DT, which maintained clearly lower variability (~0.002–0.003). For Precision SD, KNN and DT initially showed slightly higher fluctuations (~0.0045–0.0058), while NB maintained the lowest (~0.0028–0.0038) and RF remained in the stable lower range. Finally, in Recall SD, SVM and RF recorded the highest variability (~0.0055–0.0068), whereas KNN exhibited the most stable performance (~0.003–0.0035).

Overall, DT and RF demonstrated the highest stability across evaluation metrics, as indicated by their consistently low and flat SD trends. DT achieved the lowest AUC and F1 score SD values with minimal variation over time, while RF maintained the smallest Accuracy SD and exhibited stable Precision and F1 score SD values. Although RF recorded slightly higher Recall SD, its temporal fluctuation remained limited. These observations suggest that both DT and RF are less sensitive to performance variability over repeated runs, making them the most stable classifiers in this comparison.

### Validation of the Proposed Solution in a Single Topology Network

Single topology is the default configuration in Mininet, but the number of hosts can be specified.

### Scenario 1: Low-Rate DDoS Attack Running on the Single Topology

**Fig 12:**
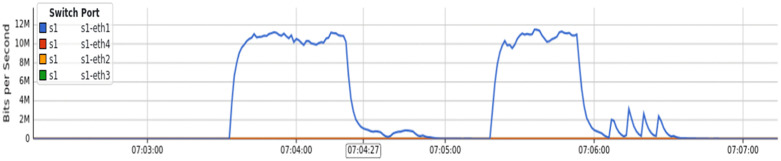
Detection of a low-rate UDP attack from node 2 to node 1 in a single topology.

Since the DT has demonstrated the lowest error rate in detecting low-rate attacks, we integrated the DT classifier into the controller. The parameters used for detecting low-rate attacks were detailed in the methodology chapter, resulting in 99.96% accuracy. As depicted in Fig. 12, we configured a single topology with one switch (s1) and four devices.

The attacker device (Node1) was connected to Ethernet port 1 (s1-eth1), while the victim device (Node2) was connected to Ethernet port 2 (s1-eth2). We initiated a low-rate UDP traffic attack (11 MB/second) from Node 1 to Node 2 using a random source technique from the hping3 tool. The controller successfully blocked all attack traffic, and the victim device remained unaffected.

### Scenario 2: High-Rate DDoS Attack Running on the Single Topology

**Fig 13:**
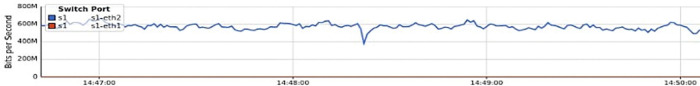
Detection of the high-rate UDP attack from node 2 to node 1 in a single topology.

Next, we integrated the RF classifier into the controller to validate our proposed method for detecting high-rate attacks. The methodology chapter detailed the parameters used for detecting low-rate attacks, resulting in 99.97% accuracy. We initiate an attack from Node 2 to Node 1, with a rate of 600 megabytes per second. Fig. 13 illustrates that the controller successfully detects and entirely blocks the attack.

### Validation of the proposed method in a fat-tree topology network

DDoS attacks, which utilize multiple attack botnets to overwhelm a target, can present challenges to mitigation techniques that focus solely on a single attack pathway. The Fat-Tree topology, renowned for its multipath capabilities, has recently gained widespread adoption in various SDN architectures [50]. Since data centers commonly implement a Fat-Tree topology architecture, we chose to leverage it to enhance the reliability of our experiment and validate the effectiveness of our model on a complex topology.

### Scenario 1: Low-Rate DDoS attack running on the fat-tree topology

**Fig 14:**
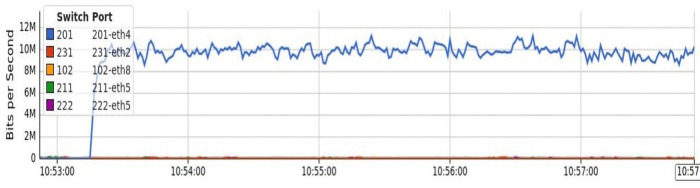
Detection of a low-rate TCP attack from node 2 to node 1 in a fat-tree topology.

The results section demonstrates that DT is more effective at detecting low-rate attacks. We embedded the DT classifier in the controller and then conducted a 10-megabyte TCP attack to validate the efficacy of our approach against different types of DDoS attacks. As depicted in Fig 14, we utilized the ‘--rand source’ command to generate random spoofed IP addresses and used them to attack the Host connected to Switch 231 through Ethernet port 2 (Port name = 231-eth2).

### Scenario 2: High-Rate DDoS Attack Running on the Fat-Tree Topology

**Fig 15:**
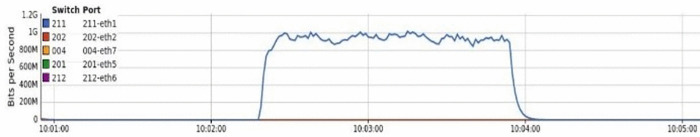
Detection of a high-rate TCP attack from node 2 to node 1 in a fat tree topology.

To validate the proposed method for detecting and mitigating high-rate TCP attacks, we embedded the RF classifier in the controller. We launched an attack from Node2 to Node1, flooding all available bandwidth with a TCP flood attack, sending 1 Gigabyte per second. The ‘--rand source’ command was used to randomly generate spoofed IP addresses and attack the Host connected to Switch 202 through Ethernet port 5 (Port Name = 202-eth2). Fig 15 illustrates that the victim’s device responded to the attack. However, the controller successfully detected and blocked the attack.

To provide a clear overview of each scenario, Table 3 summarizes the parameters for each scenario in the experiments.

**Table 3:** Experiment parameter comparison.

Scenario Description	Topology Type	Classifier Used	Attack Type	Attack Rate	Network Devices Involved	Controller Action
Low-Rate DDoS Attack (Single Topology)	Single	Decision Tree	Low-Rate	11 MB/s	Attacker (Node 1), Victim (Node 2)	Block
High-Rate DDoS Attack (Single Topology)	Single	Random Forest	High-Rate	600 MB/s	Attacker (Node 2), Victim (Node 1)	Block
Low-Rate DDoS Attack (Fat-Tree Topology)	Fat-Tree	Decision Tree	Low-Rate	10 MB/s	Attacker (Node 2), Victim (Node 1)	Block
High-Rate DDoS Attack (Fat-Tree Topology)	Fat-Tree	Random Forest	High-Rate	1 GB/s	Attacker (Node 2), Victim (Node 1)	Block

### Validation of the proposed method in a fat-tree topology network

The energy consumption resulting from the DDoS attack flood significantly decreased. The following section demonstrates this by monitoring central processing unit (CPU) usage, load, memory, and energy consumption before and after the controller is activated.

The amount of energy consumed is directly related to the CPU usage [51]. Moreover, there is a positive correlation between memory usage and energy consumption, as it relates to the number of memory accesses. In other words, the more memory is accessed, the more energy is expended. Therefore, we compared the energy consumed before and after adding the security controller to illustrate the solution’s effectiveness in reducing CPU usage and memory consumption.

**Fig 16:**
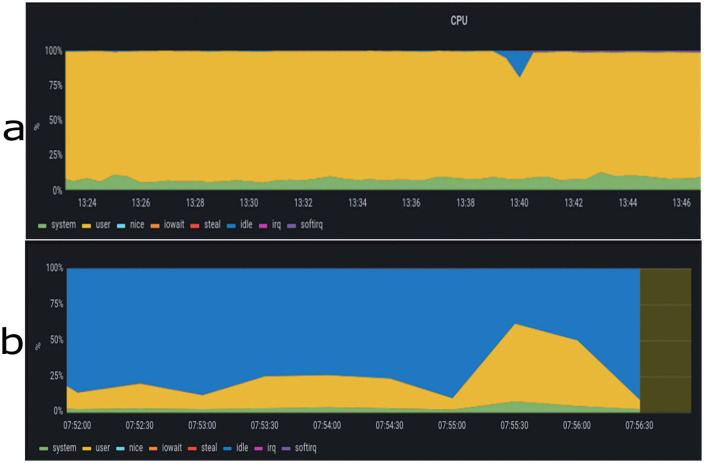
Comparison of CPU usage with and without security controller activation. (a) without security, (b) With security.

The security module operates within the controller; thus, disabling the controller means the system operates without our security module. CPU usage is a percentage value, indicating the time the CPU is actively processing tasks. Fig 16a displays the CPU state when the controller is off, showing nearly full CPU utilization during the attack. Conversely, Fig 16b illustrates the processor’s state when the controller is activated.

**Fig 17:**
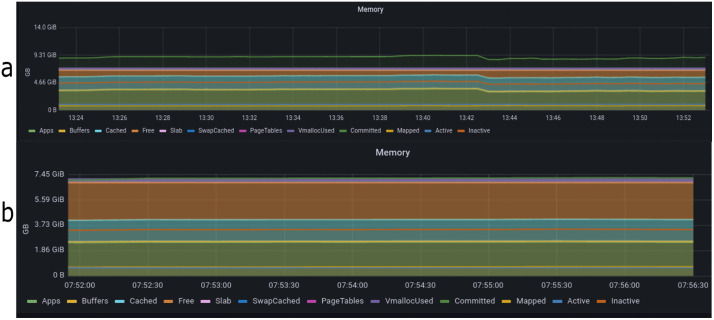
Comparison of memory consumption with and without security controller activation. (a) Without security, (b) With security.

Memory consumption reflects the amount of memory utilized during the attack period. Notably, the attack consumes a significant portion of memory, leaving minimal free memory space accessible for other purposes. Fig 17a depicts memory consumption when the controller is deactivated, whereas Fig 17b demonstrates memory consumption when the controller is activated.

**Fig 18:**
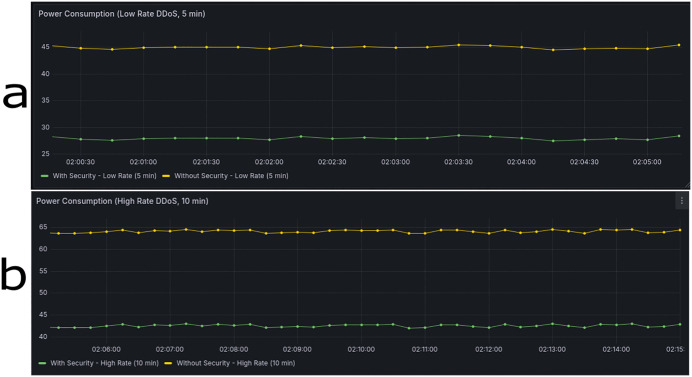
Energy Efficiency of SD-WAN Controller During DDoS Attack Scenarios. (a) Low-rate DDoS attack over 5 minutes, (b) High-rate DDoS attack over 10 minutes.

Reduced energy consumption during DDoS mitigation is quantified from CPU and memory usage trends and translated into system-level power measurements. For a low-rate DDoS attack over 5 minutes (Fig 18a), power usage with the SD-WAN controller is 28 W compared to 45 W without it, corresponding to a 37.8% reduction. For a high-rate DDoS attack over 10 minutes (Fig 18b), power consumption with the controller is 42 W versus 64 W without, representing a 34.4% reduction. These measurements were averaged over repeated simulation runs to normalize temporal fluctuations. Additionally, control scenarios across multiple attack durations (5 and 10 minutes) and attack types (low-rate and high-rate) were included to systematically evaluate energy impact. Collectively, these results demonstrate that the SD-WAN controller effectively reduces power consumption during DDoS mitigation, providing quantitative evidence of operational efficiency while maintaining robust network security.

To provide a comprehensive assessment, we analyze the performance of our proposed algorithm alongside related works mentioned earlier in this paper. S1 Table offers a comparison of detection accuracy and training time among these approaches.

The DLADSC approach by Mansoor et al. [30] achieves an accuracy of 94.18% using an RNN-based model, focusing on high accuracy but lacking precision and recall metrics. Chouhan et al. [31] present a feature extraction method utilizing SVM, RF, KNN, XGBoost, and NB algorithms, achieving an impressive accuracy of 99.39%. However, details on precision and recall for this approach are not provided. Halman and Alenazi [3 2] propose a threshold-based classifier (TBDC), achieving an accuracy of 99%, with precision, recall, and F1 score not reported.

Fotse et al. [33] achieved 98.55% accuracy using Federated Learning on CICDoS2017, CICDoS2019, and InSDN but did not provide training/testing times. Jafarian et al. [34] reached 98.8% accuracy with GBT on NetFlow + OpenFlow, yet lacked training/testing time details. Mehmood et al. [35] attained 99.95% accuracy with CNN-MLP on CICDoS2019 and InSDN but did not report training/testing time or specify the controller used. Wang et al. [36] achieved 99.24% accuracy with a CNN-based approach on InSDN but similarly did not provide training/testing time or specify the controller.

Our module consistently demonstrates high performance across multiple classification algorithms, with DT and RF achieving accuracy rates of 99.96% and 99.97%, respectively. These results underscore the effectiveness of ensemble-based methods for network traffic classification. In addition, the proposed framework employs RF-based feature extraction, achieving perfect precision, recall, and F1 score, and reliably detecting both low-rate and high-rate DDoS attacks. As illustrated in S9 Table, this explicit capability differentiates our approach from many prior ML/DL-based methods, which often report high aggregate accuracy without separately evaluating low-rate attack scenarios. The findings indicate that the proposed framework delivers both high accuracy and robustness across varying attack intensities while maintaining low computational overhead suitable for real-time SDN environments.

Additionally, our methodology benefits from a significantly larger dataset comprising 232,714 records, indicating robustness and scalability. Moreover, integrating PCA and QT techniques enhances model efficiency and effectiveness. QT-PCA facilitates dimensionality reduction by extracting relevant features from high-dimensional data, while QA optimizes model parameters and hyperparameters. This symbiotic integration of classical machine learning algorithms with quantum-inspired methodologies not only reinforces the adeptness of our module in handling complex datasets but also underscores its suitability for a wide range of classification tasks, rendering it an invaluable asset in data analysis and decision-making endeavors.

### Deployment challenges and practical considerations

Integrating the proposed detection framework within real-world SD-WAN environments presents several challenges, particularly in heterogeneous multi-vendor setups. Differences in telemetry formats, API standards, and control plane visibility across vendors necessitate the development of vendor-specific adaptation layers or middleware to ensure seamless interoperability. Additionally, resource constraints at the network edge—such as limited compute capacity and memory—require careful management to maintain low-latency detection performance. Techniques including hardware acceleration (e.g., GPU or FPGA inference via TensorRT or ONNX Runtime), model pruning, and adaptive sampling can help reduce processing overhead without sacrificing accuracy. To scale effectively, distributed or hierarchical detection architectures are recommended, wherein lightweight edge agents perform local inference and synchronize periodically with a central model, enabling collaborative detection while alleviating load on individual devices. As quantified in S3 Table, while SYN/UDP floods in our dataset closely match real-world attacks (similarity: High), ICMP and multi-vector scenarios show moderate-to-low fidelity, potentially limiting generalizability for these variants.

Although our current evaluation employs synthetic and publicly available datasets, future work involving real traffic data will adhere strictly to privacy regulations (such as GDPR and CCPA), including appropriate anonymization, synchronization and Institutional Review Board (IRB) approvals. Ethical considerations were also accounted for; no real user data was collected, and all traffic simulations were synthetically generated or sourced from open-access datasets without personally identifiable information, ensuring compliance with ethical standards. Future work should explore integrating online learning techniques such as incremental model updates and streaming algorithms to enhance adaptability in dynamic environments. Incorporating drift detection methods and hybrid detection architectures can enable the system to respond promptly to changes in network behavior and evolving attack patterns, thereby improving real-time detection capabilities. These enhancements underscore the practical deployment considerations and our commitment to addressing them in future research.

## Conclusions

In conclusion, our research presents a tailored DDoS detection solution for SDN-enabled SD-WAN environments. Our contribution lies in the engineering integration of adaptive dual-model detection framework, traffic preprocessing, and mitigation into a cohesive, testable pipeline. By leveraging the dynamic nature of SD-WAN, we can gain granular traffic visibility and detect anomalies indicative of DDoS attacks. We utilize key features such as packet count, byte count, and flow duration, which allow us to effectively identify and mitigate low-rate and high-rate attacks, even in fluctuating traffic conditions and diverse connection types.

Our approach is scalable, adaptable, and integrated with SDN controllers, enabling dynamic adjustment of detection thresholds and mitigation strategies. This facilitates protection across geographically dispersed SD-WAN sites, while the rapid incident response and coordinated mitigation enhance the network’s overall security. The experimental validation conducted within a Fat-Tree topology using Mininet demonstrates the practical applicability of our solution, confirming its effectiveness in detecting and mitigating DDoS attacks.

While our study demonstrates promising results, several important limitations must be acknowledged. First, the dataset consists primarily of synthetic traffic representing specific DDoS attack types and patterns, which may introduce bias and limit generalizability to more diverse and unpredictable real-world threat landscapes. Second, validation was conducted in a virtualized SD-WAN environment under controlled attack scenarios. Consequently, the results may not fully reflect the operational complexity, traffic heterogeneity, and dynamic conditions encountered in production deployments. To address these limitations, future research will focus on validation using real-world, heterogeneous traffic datasets, including multi-vendor SD-WAN environments with diverse application workloads and background traffic patterns. Particular emphasis will be placed on evaluating performance under mixed benign–malicious conditions and geographically distributed deployments.

A primary research direction is the incorporation of online adaptive learning and concept drift detection to maintain detection accuracy as network behavior evolves. Incremental learning techniques will enable continuous model updates using newly observed traffic, while statistical drift detection mechanisms will trigger retraining when significant distributional changes occur. Given the widespread adoption of encryption in SD-WAN communications, future work will also investigate detection of encrypted and obfuscated traffic using side-channel features that remain observable without payload inspection, such as packet size distributions, flow timing characteristics, and burst patterns.

Another critical focus is the evaluation of the framework against advanced low-and-slow attack strategies, including pulsing attacks, application-layer resource exhaustion techniques, and adaptive adversaries that modulate attack intensity to evade traditional detection thresholds. These scenarios represent realistic threats that can degrade network performance without generating obvious volumetric signatures. To improve practical deployment readiness, future work will extend the current system toward cross-domain validation across multiple SD-WAN architectures, vendors, and network topologies. This will help assess scalability, robustness, and interoperability under real operational constraints.

Beyond detection, enhanced mitigation capabilities will be developed to support dynamic and distributed defense. Planned improvements include automated attack source traceback, adaptive traffic shaping and rate limiting based on service-level objectives, and collaborative mitigation across distributed controllers. Policy orchestration mechanisms will enable mitigation responses to adjust automatically according to attack severity and network conditions. From an algorithmic perspective, further optimization will explore hybrid evolutionary approaches for feature selection and sensitivity analysis to improve model interpretability and adaptability. Deployment challenges such as integration complexity, latency constraints, and limited edge-node resources will be addressed through collaborative defense mechanisms, hardware acceleration (e.g., GPUs or FPGAs), and adaptive inference strategies. Field trials in operational environments will ultimately be essential to validate scalability, robustness, and real-world feasibility. Additionally, online learning and adaptive inference will be investigated to ensure sustained effectiveness against evolving threats in non-stationary network conditions.

In summary, this work introduces a comprehensive DDoS detection and mitigation framework tailored for SDN-enabled SD-WAN environments, combining QT-PCA preprocessing, adaptive machine learning–based detection, and integrated mitigation mechanisms. Future efforts will focus on strengthening real-world applicability, expanding coverage to emerging cyber threats, and enhancing adaptive defense capabilities to maintain robust protection across diverse deployment scenarios.

## Supporting information

S1 Table Classification results of DDoS traffic using various SDN datasets. (DOCX)

S2 Table Parameters for machine learning algorithms. (DOCX)

S3 Table Comparison of Simulated Attack Patterns and Real-World DDoS Profiles. (DOCX)

S4 Table List of Features Retained After Feature Importance and Correlation Filtering. (DOCX)

S5 Table Statistical Analysis of AUC, Accuracy, Recall, Precision, and F1 Differences Across Models. (DOCX)

S6 Table Numerical Confusion Matrices for Proposed ML Models. (DOCX)

S7 Table Ablation Study: Impact of Preprocessing Pipelines on Model. (DOCX)

S8 Table Original Network Traffic Features, Selected Features, and Their Relative Importance. (DOCX)

S9 Table Comparison of ML/DL-Based DDoS Detection Methods in SDN Environments. (DOCX)
