# Supplementary material for: Distributed denial of service detection and mitigation in software-defined networking-enabled software-defined wide area networks
Source: PLoS One. 2026 May 12;21(5):e0346673. doi: 10.1371/journal.pone.0346673 (PMC13166937; doi:10.1371/journal.pone.0346673)
Supplement: S1 Table — (DOCX) [file pone.0346673.s001.docx]

**Table S1. Classification results of DDoS traffic using various SDN datasets.**

| **Dataset description** | **PR** | **RC** | **F1** | **Dataset**  **Total Number of Records** | **Train Time/ S** | **Accuracy %** | **Algorithm** | **Consider both low and high-rate attacks.** | **Controller** | **Source** |  |
| --- | --- | --- | --- | --- | --- | --- | --- | --- | --- | --- | --- |
| DLADSC | 92.14% | N/A | 94.27% | 175,305 | 1.68 | 94.18% | RNN-based Model | No | Ryu Controller | Mansoor et al. [30] |  |
| Feature  Extraction | | 99.41% | 99.39% | 99.4% | 2850 | 0.25 | 99.39% | SVM, RF, KNN, XGBoost, NB | No | Ryu  Controller | Chouhan et al. [31] |
| TBDC | | N/A | N/A | N/A | 265 | N/A | 99% | Threshold-based Classifier | No | Ryu Controller | Halman and Alenazi [32] |
| CICDoS2017,  CICDoS2019 and InSDN | 98.21% | 98.31% | 98.51% | 343,939 | N/A | 98.55% | Federated Learning | No | Ryu Controller | Fotse et al.[33] |  |
| NetFlow + OpenFlow | 99.79% | 98.25% | 98.99% | 157,500 | N/A | 98.8% | GBTs | No | Floodlight | Jafarian et al. [34] |  |
|  |  |  |  |  |  |  |  |  |  |  |  |
| CICDoS2019,  InSDN | 99.90% | 99.97% | 99.93% | N/A | N/A | 99.95% | CNN-MLP | No | N/A | Mehmood et al.[35] |  |
| InSDN | 98.00% | 99.68%, | 99.33% | 240,710 | N/A | 99.24%, | CNN | No | N/A | Wang et al [36] |  |
| **Feature Extraction** | **100** | **100** | **100** | **232,714** | **7** | **99.97** | **DT, KNN, NP, RF, and SVM** | **Yes** | **Ryu Controller** | **Proposed method** |  |
