# Supplementary material for: Distributed denial of service detection and mitigation in software-defined networking-enabled software-defined wide area networks
Source: PLoS One. 2026 May 12;21(5):e0346673. doi: 10.1371/journal.pone.0346673 (PMC13166937; doi:10.1371/journal.pone.0346673)
Supplement: S2 Table — (DOCX) [file pone.0346673.s002.docx]

**Table S2. Default parameters were used for initial model evaluation. Final deployed models used optimized hyperparameters (see Methods).**

| ML Algorithm | Parameters | Impact on Performance |
| --- | --- | --- |
| DT | Criterion: entropy min_samples_split: 2 min_samples_leaf: 1 Random state: 0 | Utilizing the entropy criterion optimizes the model's decision-making process by selecting splits that yield the highest information gain. These values effectively mitigate overfitting while maintaining predictive accuracy. |
| KNN | n_neighbors: 5 metric: Minkowski p: 2 | The choice of 5 neighbors balances bias and variance, allowing the model to generalize well to unseen data. Minkowski distance accurately captures the distribution of data points in the feature space. |
| SVM | Kernel: Radial Basis Function (RBF) Random state: 0 | The RBF kernel is adept at handling non-linear data, which is crucial for effectively capturing complex patterns within network traffic. Ensures robust classification even in high-dimensional spaces. |
| RF | n_estimators: 10 Criterion: entropy  Random state: 0 | A limited number of trees (10) reduces computational load while leveraging ensemble learning benefits. Entropy as a criterion helps maintain consistent performance across varying datasets. |
| NB | Priors: None var_smoothing: 1e-09 | Allowing the model to learn from the data without imposing prior assumptions enhances flexibility. The small value of var_smoothing ensures numerical stability, particularly when dealing with small feature variances. |
