# Supplementary material for: Distributed denial of service detection and mitigation in software-defined networking-enabled software-defined wide area networks
Source: PLoS One. 2026 May 12;21(5):e0346673. doi: 10.1371/journal.pone.0346673 (PMC13166937; doi:10.1371/journal.pone.0346673)
Supplement: S3 Table — (DOCX) [file pone.0346673.s003.docx]

**Table S3. Comparison of Simulated Attack Patterns and Real-World DDoS Profiles.**

| Attack Vector | Simulated Dataset Characteristics | Real-World Dataset Characteristics (CICDDoS2019 / inSDN) | Similarity Level |
| --- | --- | --- | --- |
| SYN Flood | Sustained TCP SYN packets to port 80, fixed packet size (64 bytes), constant high rate (1k–10k pps) | Variable packet sizes, randomized destination ports, adaptive rate (1k–25k pps), often part of a multi-vector attack | High |
| UDP Flood | High-rate floods using random source IPs, constant rate (1k–5k pps), standard UDP headers | Mixed-rate traffic, spoofed IPs, randomized packet sizes and payloads (64–1500 bytes), often bursty | Moderate |
| HTTP Flood | GET requests to static URLs with fixed headers, sustained connection durations | Randomized HTTP headers and URIs, variable connection timing, slow-rate and stealth variants are included | Moderate |
| ICMP Flood (inSDN) | Ping floods with constant intervals and fixed payload | ICMP floods include both echo-request/echo-reply variants, randomized timing, embedded in hybrid attack scenarios | Low to Moderate |
| Multi-vector | Not included | Attacks often combine SYN/UDP/ICMP with shifting vectors to evade detection | Not Represented |
| Time-varying behavior | Fixed durations, simple schedule (attack vs. benign phases) | Variable-length attacks, dynamic switching, gradual ramp-up phases | Limited |
