# Supplementary material for: Distributed denial of service detection and mitigation in software-defined networking-enabled software-defined wide area networks
Source: PLoS One. 2026 May 12;21(5):e0346673. doi: 10.1371/journal.pone.0346673 (PMC13166937; doi:10.1371/journal.pone.0346673)
Supplement: S4 Table — (DOCX) [file pone.0346673.s004.docx]

**Table S4. List of Features Retained After Feature Importance and Correlation Filtering.**

| Feature Name | Category | Description |
| --- | --- | --- |
| kt_count | Flow Statistic | Total number of packets received |
| byte_count | Flow Statistic | Total number of bytes received |
| flow_dur_sec | Flow Statistic | Duration of the flow in seconds |
| flow_dur_nsec | Flow Statistic | Duration of the flow in nanoseconds |
| pkt_count_sec | Flow Statistic | Number of packets per second |
| byte_count_sec | Flow Statistic | Number of bytes per second |
| flags | Flow Metadata | Indicates physical port behaviour |
| ip_proto | Network Layer | IP protocol type (TCP, UDP, ICMP) |
| icmp_type | Transport Layer | ICMP message type |
| icmp_code | Transport Layer | ICMP message code |
| idl_time_out | Flow Timeout Config | Idle timeout value of the flow entry |
| hd_time_out | Flow Timeout Config | Hard timeout value of the flow entry |
| ip_source | Network Layer | Source IP address |
| ip_dstn | Network Layer | Destination IP address |
| tp_dstn | Transport Layer | Destination port |
