# Supplementary material for: Distributed denial of service detection and mitigation in software-defined networking-enabled software-defined wide area networks
Source: PLoS One. 2026 May 12;21(5):e0346673. doi: 10.1371/journal.pone.0346673 (PMC13166937; doi:10.1371/journal.pone.0346673)
Supplement: S5 Table — (DOCX) [file pone.0346673.s005.docx]

**S5 Table. Statistical Analysis of AUC, Accuracy, Recall, Precision, and F1 Score Differences Across Models.**

| Model 1 | Model 2 | Mean AUC Diff (±σ) | Mean Acc Diff (±σ) | Mean Recall Diff (±σ) | Mean Precision Diff (±σ) | Mean F1 Score Diff (±σ) | p-value (AUC) | Significant? | Test Used |
| --- | --- | --- | --- | --- | --- | --- | --- | --- | --- |
| Random Forest | Decision Tree | +0.0004 (±0.0002) | +0.0003 (±0.0001) | +0.0005 (±0.0003) | +0.0005 (±0.0002) | +0.0004 (±0.0002) | 0.038 | Yes | Paired t-test |
| Random Forest | SVM | +0.0014 (±0.0005) | +0.0012 (±0.0003) | +0.0015 (±0.0006) | +0.0015 (±0.0004) | +0.0013 (±0.0003) | 0.002 | Yes | Paired t-test |
| Random Forest | Naive Bayes | +0.0881 (±0.0051) | +0.0942 (±0.0048) | +0.1054 (±0.0062) | +0.0920 (±0.0053) | +0.0643 (±0.0042) | <0.001 | Yes | Wilcoxon |
| Decision Tree | SVM | +0.0010 (±0.0004) | +0.0009 (±0.0002) | +0.0010 (±0.0005) | +0.0010 (±0.0003) | +0.0009 (±0.0002) | 0.012 | Yes | Paired t-test |
| Decision Tree | KNN | +0.0149 (±0.0012) | +0.0142 (±0.0010) | +0.0158 (±0.0015) | +0.0135 (±0.0011) | +0.0140 (±0.0010) | <0.001 | Yes | Paired t-test |
| SVM | Naive Bayes | +0.0867 (±0.0050) | +0.0930 (±0.0047) | +0.1039 (±0.0060) | +0.0905 (±0.0051) | +0.0630 (±0.0040) | <0.001 | Yes | Wilcoxon |
