# Supplementary material for: Distributed denial of service detection and mitigation in software-defined networking-enabled software-defined wide area networks
Source: PLoS One. 2026 May 12;21(5):e0346673. doi: 10.1371/journal.pone.0346673 (PMC13166937; doi:10.1371/journal.pone.0346673)
Supplement: S6 Table — (DOCX) [file pone.0346673.s006.docx]

**S6 Table. Numerical Confusion Matrices for Proposed ML Models**

| Model | +True Label / Predicted Label | Normal Traffic | Low-rate Attack | High-rate Attack |
| --- | --- | --- | --- | --- |
| SVM (a) | Normal Traffic | 12 556 | 0 | 0 |
|  | Low-rate Attack | 5 | 20 892 | 1 |
|  | High-rate Attack | 83 | 1 089 | 23 553 |
| RF (b) | Normal Traffic | 12 555 | 0 | 1 |
|  | Low-rate Attack | 1 | 20 898 | 0 |
|  | High-rate Attack | 1 | 15 | 24 709 |
| KNN (c) | Normal Traffic | 12 553 | 0 | 3 |
|  | Low-rate Attack | 5 | 20 784 | 109 |
|  | High-rate Attack | 29 | 723 | 29 373 |
| NB (d) | Normal Traffic | 12 133 | 0 | 423 |
|  | Low-rate Attack | 876 | 15 722 | 671 |
|  | High-rate Attack | 1 481 | 3 258 | 20 668 |
| DT (e) | Normal Traffic | 12 556 | 0 | 0 |
|  | Low-rate Attack | 0 | 20 889 | 9 |
|  | High-rate Attack | 0 | 14 | 24 711 |
