# Supplementary material for: Distributed denial of service detection and mitigation in software-defined networking-enabled software-defined wide area networks
Source: PLoS One. 2026 May 12;21(5):e0346673. doi: 10.1371/journal.pone.0346673 (PMC13166937; doi:10.1371/journal.pone.0346673)
Supplement: S7 Table — (DOCX) [file pone.0346673.s007.docx]

**S7 Table. Ablation Study: Impact of Preprocessing Pipelines on Model.**

| Preprocessing Configuration | Description | F1-Score（%） | Accuracy（%） | Training Time (s) | Inference Time (ms/sample) |
| --- | --- | --- | --- | --- | --- |
| Raw | No preprocessing applied. | 85.40 | 86.92 | 125 | ~12 |
| PCA Only | PCA applied to raw, unscaled features. | 90.67 | 92.80 | 95 | ~8 |
| QT Only | QT applied, all features retained. | 95.85 | 95.50 | 150 | ~15 |
| QT→PCA (Proposed) | QT applied, then PCA for dimensionality reduction. | 99.99 | 99.97 | 7 | <5 |
