# Supplementary material for: Distributed denial of service detection and mitigation in software-defined networking-enabled software-defined wide area networks
Source: PLoS One. 2026 May 12;21(5):e0346673. doi: 10.1371/journal.pone.0346673 (PMC13166937; doi:10.1371/journal.pone.0346673)
Supplement: S8 Table — (DOCX) [file pone.0346673.s008.docx]

**S8 Table. Original Network Traffic Features, Selected Features, and Their Relative Importance.**

| Feature Name | Description | Selected | Relative Importance |
| --- | --- | --- | --- |
| time_stamp | Current timestamp for an event | Yes | 0.031 |
| data_path_id | Datapath ID for the arriving traffic | Yes | 0.027 |
| flow_id | Unique ID for arriving traffic | Yes | 0.029 |
| ip_source | Packet source IP address | Yes | 0.058 |
| tp_source | Packet source port address | Yes | 0.052 |
| ip_dstn | Destination IP address | Yes | 0.056 |
| tp_dstn | Destination port address | Yes | 0.049 |
| ip_proto | IP protocol type | Yes | 0.063 |
| icmp_code | First byte from the ICMP message | Yes | 0.022 |
| icmp_type | Second byte from the ICMP message | Yes | 0.024 |
| flow_dur_sec | Flow duration in seconds | Yes | 0.061 |
| flow_dur_nsec | Flow duration in nanoseconds | Yes | 0.047 |
| idl_time_out | Entry validity timeout (seconds) | Yes | 0.036 |
| hd_time_out | Absolute timeout after blocking a flow | Yes | 0.034 |
| flags | Physical port behavior indicators | Yes | 0.041 |
| pkt_count | Packet arrival rate | Yes | 0.072 |
| byte_count | Byte arrival rate | Yes | 0.069 |
| pkt_count_sec | Packets per second | Yes | 0.081 |
| pkt_count_nsec | Packets per nanosecond | Yes | 0.038 |
| byte_count_sec | Bytes per second | Yes | 0.076 |
| byte_count_nsec | Bytes per nanosecond | Yes | 0.034 |
| flow_priority | Flow priority level | No | 0.009 |
| table_id | OpenFlow table identifier | No | 0.007 |
| idle_age | Time since last packet match | No | 0.011 |
| hard_age | Time since rule installation | No | 0.010 |
| packet_in_count | Number of PACKET_IN events | No | 0.014 |
| port_no | Switch port number | No | 0.008 |
| queue_id | Queue identifier | No | 0.006 |
| vlan_id | VLAN identifier | No | 0.012 |
| tos | Type of Service field | No | 0.013 |
| tcp_flags | TCP flag combination | No | 0.015 |
| rx_bytes | Received bytes at port | No | 0.010 |
| tx_bytes | Transmitted bytes at port | No | 0.009 |
| duration | Overall flow duration | No | 0.016 |
