# Supplementary material for: Distributed denial of service detection and mitigation in software-defined networking-enabled software-defined wide area networks
Source: PLoS One. 2026 May 12;21(5):e0346673. doi: 10.1371/journal.pone.0346673 (PMC13166937; doi:10.1371/journal.pone.0346673)
Supplement: S9 Table — (DOCX) [file pone.0346673.s009.docx]

**S9 Table. Comparison of ML/DL-Based DDoS Detection Methods in SDN Environments.**

| Study | Method | Accuracy | Precision | Recall | F1 Score | Low-Rate Detection Capability | High-Rate Detection Capability | Mitigation capability | Recourse overhead | |
| --- | --- | --- | --- | --- | --- | --- | --- | --- | --- | --- |
|  |  |  |  |  |  |  |  |  | **CPU Usage** | **Inference time** |
| Mansoor et al. [30] | RNN | 94.18% | 92.14% | 96.50% | 94.27% | No | Limited (volume-dependent) | No | NA | NA |
| Chouhan et al. [31] | SVM/RF/ KNN/ XGB | 99.39% | 99.41% | 99.39% | 99.40% | No | Yes | Yes | NA | NA |
| Fotse et al. [33] | Federated Learning | 98.55% | 98.21% | 98.31% | 98.51% | No | Yes | Yes | NA | NA |
| Jafarian et al. [34] | Gradient Boosting Trees | 98.80% | 99.79% | 98.25% | 98.99% | No | Yes | Yes | NA | NA |
| Mehmood et al. [35] | CNN-MLP | 99.95% | 99.90% | 99.97% | 99.93% | No | Yes | No | NA | NA |
| Wang et al. [36] | CNN + Wavelet | 99.24% | 98.00% | 99.68% | 99.33% | No | Yes | Yes | NA | NA |
| Proposed Framework | Adaptive RF/DT | 99.97% | 99.99% | 99.99% | 99.99% | Yes (99.95%) | Yes | Yes | 12.9% (DT) | <5 MS (DT) |
|  |  |  |  |  |  |  |  |  | 13.8% (RF) | ~10 MS (RF) |
